# Per-Feature Accuracy of Liver Imaging Reporting and Data System Locoregional Treatment Response Algorithm: A Systematic Review and Meta-Analysis

**DOI:** 10.3390/cancers13174432

**Published:** 2021-09-02

**Authors:** Yeon Jong Huh, Dong Hwan Kim, Bohyun Kim, Joon-Il Choi, Sung Eun Rha

**Affiliations:** Department of Radiology, Seoul St. Mary’s Hospital, College of Medicine, The Catholic University of Korea, 222 Banpo-daero, Seocho-gu, Seoul 06591, Korea; nazg@catholic.ac.kr (Y.J.H.); kbh@catholic.ac.kr (B.K.); dumky@catholic.ac.kr (J.-I.C.); serha@catholic.ac.kr (S.E.R.)

**Keywords:** liver neoplasms, hepatocellular carcinoma, Liver Imaging Reporting and Data Systems (LI-RADS), treatment outcome, systematic review, meta-analysis

## Abstract

**Simple Summary:**

Locoregional therapy (LRT) is widely performed as a nonsurgical treatment for hepatocellular carcinoma (HCC). Following LRT, precise assessment of post-treatment imaging can play an important role in determining residual tumor viability and future treatment for patients with HCC. Owing to the need to provide a more standardized image interpretation, Liver Imaging Reporting and Data Systems (LI-RADS) treatment response (TR) algorithm was developed. We conducted a systematic review and meta-analysis to assess the accuracy of each imaging feature of LI-RADS TR (LR-TR) viable category for diagnosing viable HCC after LRT. This meta-analysis of 10 studies comprising 971 patients found that the pooled sensitivity and diagnostic odds ratio were the highest for arterial phase hyperenhancement (APHE), followed by washout appearance and enhancement similar to pretreatment. The diagnostic performance of APHE was significantly different depending on the type of reference standard and MRI contrast agent. The results of this meta-analysis represent the currently available evidence regarding the performance of LR-TR algorithm.

**Abstract:**

We aimed to investigate the accuracy of each imaging feature of LI-RADS treatment response (LR-TR) viable category for diagnosing tumor viability of locoregional therapy (LRT)-treated HCC. Studies evaluating the per feature accuracy of the LR-TR viable category on dynamic contrast-enhanced CT or MRI were identified in databases. A bivariate random-effects model was used to calculate the pooled sensitivity, specificity, and diagnostic odds ratio (DOR) of LR-TR viable features. Ten studies assessing the accuracies of LR-TR viable features (1153 treated observations in 971 patients) were included. The pooled sensitivities and specificities for diagnosing viable HCC were 81% (95% confidence interval [CI], 63–92%) and 95% (95% CI, 88–98%) for nodular, mass-like, or irregular thick tissue (NMLIT) with arterial phase hyperenhancement (APHE), 55% (95% CI, 34–75%) and 96% (95% CI, 94–98%) for NMLIT with washout appearance, and 21% (95% CI, 6–53%) and 98% (95% CI, 92–100%) for NMLIT with enhancement similar to pretreatment, respectively. Of these features, APHE showed the highest pooled DOR (81 [95% CI, 25–261]), followed by washout appearance (32 [95% CI, 13–82]) and enhancement similar to pretreatment (14 [95% CI, 5–39]). In conclusion, APHE provided the highest sensitivity and DOR for diagnosing viable HCC following LRT, while enhancement similar to pretreatment showed suboptimal performance.

## 1. Introduction

Locoregional therapy (LRT), including transarterial chemoembolization (TACE) and radiofrequency ablation (RFA), is widely performed as a nonsurgical treatment for patients who are not candidates for liver transplantation or surgical resection [1,2,3,4]. Patients with HCC can undergo LRT as a definite treatment for early-stage HCC or as a bridge or downstaging procedure prior to liver transplantation [1,2,3,4]. Following LRT for HCC, the treatment response is usually assessed by dynamic contrast-enhanced computed tomography (CT) or magnetic resonance imaging (MRI) as recommended by major international guidelines [2,4]. Therefore, precise and consistent assessment of post-treatment imaging can play an important role in determining residual tumor viability and future treatment for patients following LRT [5,6].

Owing to the need to provide a more standardized form of image interpretation and reporting, Liver Imaging Reporting and Data Systems (LI-RADS) introduced a treatment response algorithm in 2017 for the evaluation of treated observations after LRT [7]. The LI-RADS treatment response (LR-TR) algorithm proposed post-treatment imaging features on contrast-enhanced CT or MRI to categorize treated observations as either LR-TR viable (probably or definitely viable), LR-TR equivocal (equivocally viable), or LR-TR nonviable [7]. In particular, the imaging features suggestive of LR-TR viable are nodular, mass-like, or irregular thick tissue (NMLIT) in or along the treated lesion with any of the following: arterial phase hyperenhancement (APHE), washout appearance, or enhancement similar to pretreatment [7]. The LR-TR algorithm adds new imaging features for the viability of HCC, i.e., washout appearance and enhancement similar to pretreatment, whereas the modified Response Evaluation Criteria in Solid Tumors or European Association for the Study of the Liver criteria consider APHE to be the only characteristic of a viable tumor [8,9].

With increased attention to the LR-TR algorithm, several studies addressed the diagnostic performance of LR-TR for diagnosing viable HCC, and a recent meta-analysis indicated a sensitivity and specificity of 63% and 96% for the LR-TR viable category, respectively [10]. However, in addition to the performance of the LR-TR viable category, there is an important issue in the performance of each imaging feature of LR-TR, which was reported in a wide variety of previous studies [11,12,13,14]. Moreover, the pooled sensitivity and specificity of the newly adopted imaging features in the LR-TR algorithm, i.e., NMLIT with washout appearance and enhancement similar to pretreatment, are unknown. Therefore, we aimed to investigate the accuracy of each imaging feature of the LR-TR viable category for diagnosing the viability of HCC treated with LRT.

## 2. Materials and Methods

This meta-analysis was performed in compliance with the Preferred Reporting Items for Systematic Reviews and Meta-Analyses guidelines [15] and was prospectively registered in PROSPERO (ID: CRD42021248915). The following literature search, study selection, data extraction, and study quality assessment were independently conducted by two reviewers (each having ≥2 years of experience in liver imaging and meta-analysis), followed by discussion with a third reviewer (with 11 years of experience in liver imaging) in case of disagreement.

### 2.1. Literature Search Strategy

PubMed MEDLINE and EMBASE databases were searched to identify original research articles reporting the performance of imaging features of the LR-TR viable category for the diagnosis of viable HCC after LRT. The search queries included “Liver”, “LI-RADS”, “LI-RADS Treatment Response”, “CT”, and “MRI”, and a detailed list of the search terms is presented in the Table S1. The literature search was conducted from 1 January 2017 to 25 May 2021. The search was limited to original studies on human subjects written in English.

### 2.2. Inclusion and Exclusion Criteria

The inclusion criteria were as follows: (1) population: patients undergoing LRT for HCC; (2) index test: dynamic contrast-enhanced CT or MRI; (3) reference standard: surgical pathology or composite clinical reference standard (CCRS); (4) outcomes: diagnostic performance of the three imaging features (i.e., NMLIT with APHE, washout appearance, and enhancement similar to pretreatment) of LR-TR viable category for the diagnosis of viable HCC treated with LRT; and (5) study design: observational studies (prospective or retrospective) and clinical trials. The exclusion criteria included the following: (1) case reports, review articles, editorials, scientific abstracts, systematic reviews, and meta-analyses; (2) studies that were not within the field of interest of this study; and (3) studies without sufficient details to construct a diagnostic 2-by-2 table of the imaging results and reference standards. Articles were first screened by titles and abstracts and were fully reviewed after the first screening.

### 2.3. Data Extraction and Quality Assessment

The following data were extracted from each eligible study: (1) study characteristics regarding authors, publication year, and design (prospective or retrospective); (2) subject characteristics regarding number of patients, age, and dominant etiology of underlying liver disease; (3) number of treated observations; (4) type of LRTs performed in each study; (5) imaging modality, either CT or MRI; (6) MRI characteristics regarding type of contrast agents and MRI magnet; (7) image analysis method (multiple independent reviewers or multiple reviewers with consensus), number of reviewers, and experience level of reviewers for liver imaging; (8) reference standard for viable HCC; (9) interobserver agreement (κ) for the presence of each imaging feature of the LR-TR viable category on CT or MRI; and (10) study outcomes, i.e., the numbers of true positives, false positives, false negatives, and true negatives of each imaging feature for diagnosing viable HCC (Table S2). If not distinctly mentioned, data were manually retrieved from tables and figures. If more than one dataset was available within the study, i.e., multiple independent reviewers, the data with the highest accuracy were chosen to perform the meta-analysis. When an article did not contain sufficient data, we contacted the corresponding authors by email to request additional information or clarification.

The quality of the included articles was evaluated using the Quality Assessment of Diagnostic Accuracy Studies (QUADAS-2) tool [16], which focused on the four different domains of patient selection, index test, reference standard, and flow and timing.

### 2.4. Data Synthesis and Statistical Analysis

The unit of analysis was per observation. The sensitivity and specificity of each imaging feature of the LR-TR viable category and their 95% confidence intervals (CIs) were obtained from each study. The meta-analytic pooled sensitivity, specificity, and their 95% CIs were calculated using a bivariate random-effects and hierarchical summary receiver operating characteristic (HSROC) model. The meta-analytic pooled diagnostic odds ratio (DOR) for diagnosing viable HCC with corresponding 95% CIs was also calculated for each imaging feature using a bivariate random-effects model. Subgroup analyses according to the imaging modality (MRI versus CT) were performed and compared using joint-model bivariate meta-regression.

Heterogeneity was assessed using the Cochran’s Q test (*p* < 0.10 indicates substantial heterogeneity) and *I*^2^ statistic (*I*^2^ > 50% indicates substantial heterogeneity). The presence of a threshold effect was analyzed by the visual assessment of the coupled forest plots of sensitivity and specificity, as well as by calculating the Spearman correlation coefficient between the sensitivity and false-positive rate [17]. A correlation coefficient >0.6 was considered to indicate a considerable threshold effect [17]. When substantial heterogeneity was noted, meta-regression analysis was performed to further investigate the causes. The following covariates were considered: (1) reference standard (pathology only versus CCRS or both), (2) MRI contrast agent (hepatobiliary contrast agent [HBA] only versus extracellular contrast agent [ECA] or both), (3) type of LRT (transcatheter therapy, i.e., TACE or transarterial radioembolization, was performed in more than 70% of observations versus others), (4) image analysis method (multiple independent reviewers versus multiple reviewers with consensus), and (5) percentage of viable HCC among treated observations (≥50% versus <50%).

Deeks’ funnel plot and Deeks’ asymmetry test were used to assess the presence of publication bias. Stata version 16.0 (StataCorp LP, College Station, TX, USA) was used for statistical analysis, with *p* < 0.05 considered statistically significant.

## 3. Results

### 3.1. Literature Search

**Figure 1:**
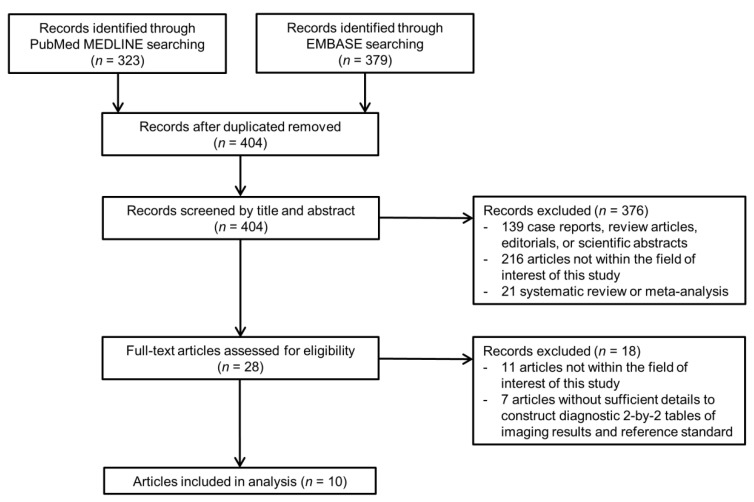
Flow diagram of article selection process.

A total of 404 articles were screened after removing duplicates (Figure 1). Of these, 376 articles were excluded based on their titles and abstracts. Eighteen articles were further excluded after a full text review. Specifically, studies that reported on the performance of LR-TR viable category but did not report data on the performance of each imaging feature were excluded [18,19,20]. Finally, a total of 10 eligible articles reported the diagnostic performance of LR-TR viable features for diagnosing viable HCC (Table 1) [11,12,13,14,21,22,23,24,25,26]. Of the 10 eligible articles, 10 reported the performance of NMLIT with APHE [11,12,13,14,21,22,23,24,25,26], 8 reported that of NMLIT with washout appearance [11,12,13,14,22,23,24,25], and 6 reported that of NMLIT with enhancement similar to pretreatment [12,13,14,22,23,25].

### 3.2. Study Characteristics

The characteristics of the finalized studies included in the meta-analysis are summarized in Table 1 (971 patients with 1,153 treated observations). Of the 10 included studies, 1 had a prospective study design [21], and 9 were of retrospective design [11,12,13,14,22,23,24,25,26]. Hepatitis B was the dominant etiology of liver disease in eight studies [11,12,13,14,22,23,24,25]. Patients were treated only with conventional TACE in two studies [13,21], transarterial radioembolization in one study [25], and ablative therapy (RFA or microwave ablation) in two studies [24,26]. Various types of LRT were performed in the remaining five studies, but conventional TACE was the most frequently performed treatment modality [11,12,14,22,23]. Five studies used only MRI [11,14,21,24,26], one used only CT [13], and four used both MRI and CT [12,22,23,25]. Two used only a 3.0-T MRI scanner [21,25], and four used only HBA (gadoxetate disodium) [11,12,14,23]. Except for one study [21], image analysis was performed by multiple reviewers. Among them, reviewers worked independently in three studies [12,13,22] and with consensus in six studies [11,14,23,24,25,26]. Five studies used only pathological diagnosis as a reference standard for viable HCC [12,14,22,23,25], while the other five used CCRS or both as reference standards [11,13,21,24,26].

### 3.3. Quality of Included Studies

The overall quality of the included studies is presented in Figure S1. In the patient selection domain, one study was at high risk for selection bias because a substantial proportion of patients (38%, 119/316) without appropriate reference standards to determine the viabilities of the treated lesions were excluded [11]. Three studies were unclear whether the index test result was interpreted without knowledge of the reference standard [21,24,26], resulting in a risk of bias in the index test domain. Three studies had a high risk of bias in the reference standard domain because they used only CCRS as a reference standard, which lacked independence between diagnostic and reference tests [21,24,26]. There was an unclear risk of bias in the flow and timing domain in three studies because the patients did not receive the same reference standard [11,13,21].

### 3.4. Diagnostic Performance of Imaging Features of the LR-TR Viable Category for Diagnosing Viable HCC

**Figure 2:**
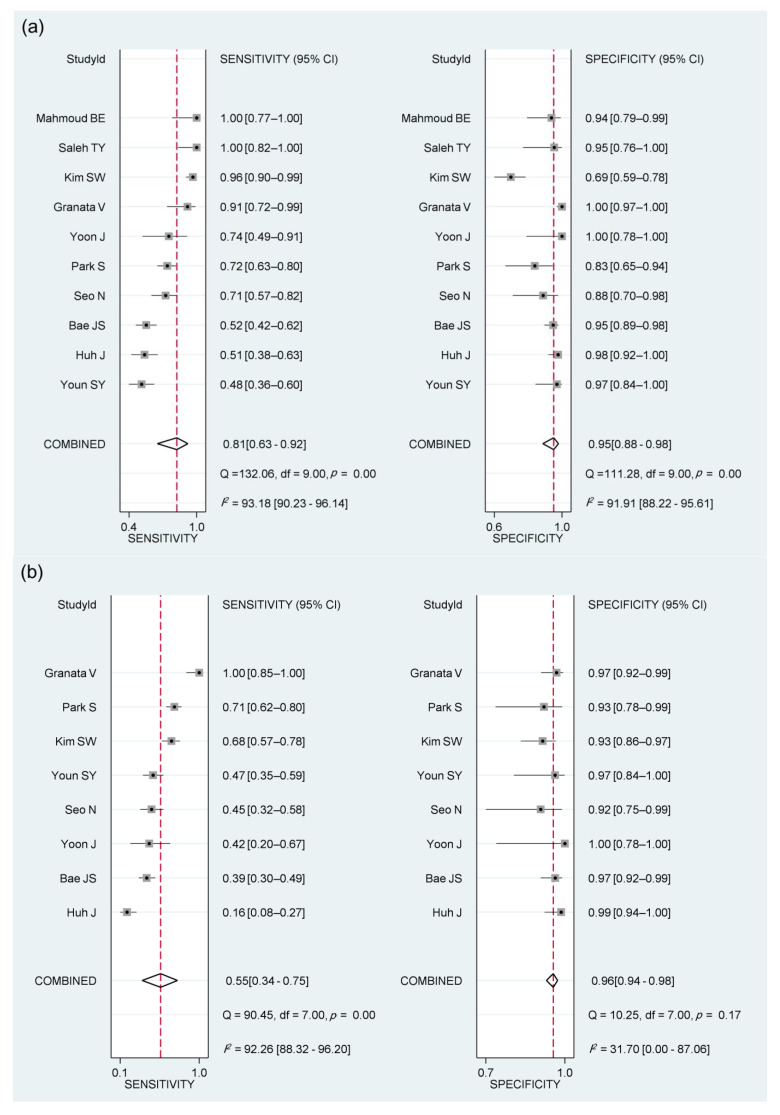

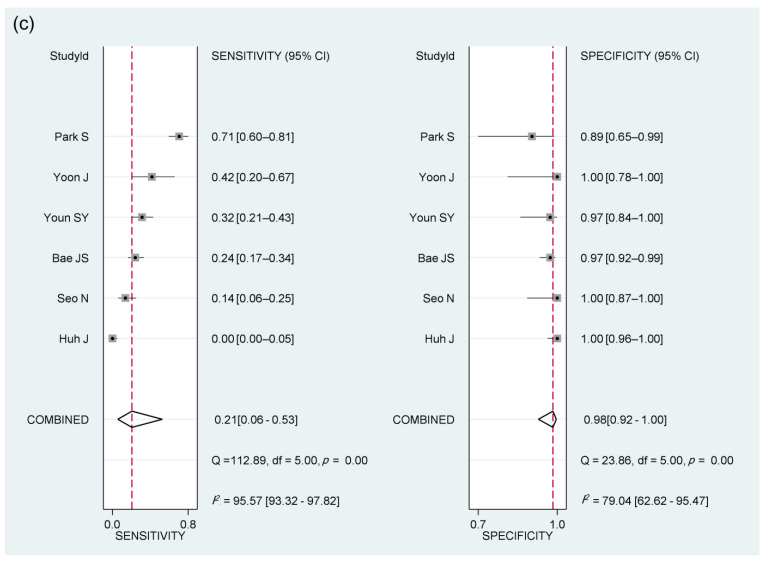
Coupled forest plots of sensitivity and specificity of arterial phase hyperenhancement (**a**), washout appearance (**b**), or enhancement similar to pretreatment (**c**) for diagnosing the viability of hepatocellular carcinoma.

**Figure 3:**
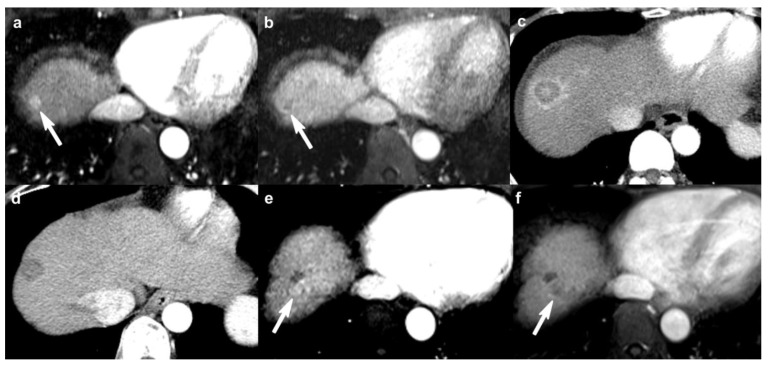
A 54-year-old man with HCC treated with RFA. (**a**,**b**) Pretreatment MR images on the arterial phase (**a**) and portal venous phase (**b**) show a 1.0-cm arterial enhancing and washout nodule on liver segment VIII dome (arrows), suggestive of HCC. (**c**) Immediate post-RFA CT image shows complete ablation of HCC, surrounded by expected post-treatment thin rim enhancement on the arterial phase. (**d**) Follow-up (after 3 months) CT image on the arterial phase shows no lesional enhancement in treated observation. This treated observation was assigned as LR-TR nonviable. (**e**,**f**) However, follow-up (after 3 years) MR images on arterial phase (**e**) and portal venous phase (**f**) show nodular arterial enhancement and washout at posterior margin of treated observation (arrows). This treated observation was assigned as LR-TR viable category and confirmed as viable HCC on explant pathology. HCC, hepatocellular carcinoma; RFA, radiofrequency ablation; MR, magnetic resonance; CT, computed tomography; LR-TR, Liver Imaging Reporting and Data System treatment response.

The pooled sensitivities and specificities for diagnosing viable HCC were 81% (95% CI, 63–92; *I*^2^, 93%; Cochran’s Q test, *p* < 0.1) and 95% (95% CI, 88–98; *I*^2^, 92%; Cochran’s Q test, *p* < 0.1), respectively, for NMLIT with APHE; 55% (95% CI, 34–75; *I*^2^, 92%; Cochran’s Q test, *p* < 0.1) and 96% (95% CI, 94–98; *I*^2^, 32%; Cochran’s Q test, *p* = 0.17), respectively, for NMLIT with washout appearance; and 21% (95% CI, 6–53; *I*^2^, 96%; Cochran’s Q test, *p* < 0.1) and 98% (95% CI, 92–100; *I*^2^, 79%; Cochran’s Q test, *p* < 0.1), respectively, for NMLIT with enhancement similar to pretreatment (Table 2, Figure 2 and Figure 3). The HSROC curves with 95% confidence and prediction regions showed a large difference between the two regions, indicating considerable heterogeneity between studies for all three imaging features (Figure S2). The area under the summary receiver operating characteristic curve was 0.96 (95% CI, 0.94–0.97) for NMLIT with APHE, 0.96 (95% CI, 0.94–0.98) for NMLIT with washout appearance, and 0.89 (95% CI, 0.86–0.92) for NMLIT with enhancement similar to pretreatment. All three imaging features were significantly associated with viable HCC, demonstrating 95% CIs of their pooled DORs not enclosing 1.0 (Table 2). Of the imaging features, NMLIT with APHE showed the highest meta-analytic pooled DOR (81 [95% CI, 25–261]), followed by washout appearance (32 [95% CI, 13–82]) and enhancement similar to pretreatment (14 [95% CI, 5–39]).

Of the three imaging features, a significant threshold effect between sensitivity and specificity was observed only in NMLIT with enhancement similar to pretreatment (Spearman correlation coefficient, 0.64; *p* = 0.17). There were no significant threshold effects for the other two features (rho ≤ 0.38; *p* ≥ 0.35). No significant publication bias was noted for any imaging features across the studies (*p* ≥ 0.15, Table 2 and Figure S3).

### 3.5. Subgroup Analyses According to Imaging Modality

In all three imaging features, the pooled sensitivities tended to be higher on MRI than on CT, although the difference was not statistically significant (*p* ≥ 0.07; Table 2). For NMLIT with APHE, the pooled sensitivity and specificity on MRI were 87% (95% CI, 67–96) and 93% (95% CI, 84–97), respectively, and those on CT were 50% (95% CI, 36–64) and 96% (95% CI, 89–98), respectively (*p* = 0.07). For NMLIT with washout appearance, the pooled sensitivity and specificity on MRI were 66% (95% CI, 41–84) and 95% (95% CI, 93–97), respectively, and those on CT were 35% (95% CI, 17–59) and 97% (95% CI, 93–99), respectively (*p* = 0.17). For NMLIT with enhancement similar to pretreatment, the pooled sensitivity and specificity on MRI were 32% (95% CI, 16–54) and 96% (95% CI, 89–99), respectively, and those on CT were 15% (95% CI, 1–68) and 99% (95% CI, 87–100), respectively (*p* = 0.78).

### 3.6. Meta-Regression Analysis

Table 3 summarizes the results of the meta-regression analysis for study heterogeneity. The type of reference standard and MRI contrast agent were significantly associated with study heterogeneity for NMLIT with APHE (*p* ≤ 0.03). Studies that used only pathology as a reference standard had significantly lower sensitivity (63% versus 91%) and comparable specificity (94% versus 95%) to that of those that used CCRS or both as reference standards. In addition, studies using HBA showed a lower sensitivity (73% versus 90%) and specificity (89% versus 97%) than that of those using ECA or both. For NMLIT with washout appearance, the image analysis method was significantly associated with heterogeneity (*p* = 0.03). Studies in which image analysis was conducted by consensus among multiple reviewers had a higher sensitivity (73% versus 34%) and a comparable specificity (95% versus 97%) to that of those conducted by multiple independent reviewers. The other covariates were not significantly associated with study heterogeneity.

### 3.7. Interobserver Agreement for Imaging Features of the LR-TR Viable Category

Three included studies reported interobserver agreements (κ) for the presence of each imaging feature of the LR-TR viable category on MRI [14,22,23], two studies reported those on CT [22,23], and one study reported those in a combination of CT and MRI [25]. For MRI, the κ values regarding NMLIT with APHE, washout, and enhancement similar to pretreatment ranged from 0.67 to 0.75, 0.52 to 0.64, and 0.41 to 0.76, respectively. For CT, the κ values regarding NMLIT with APHE, washout, and enhancement similar to pretreatment ranged from 0.71 to 0.80, 0.67 to 0.72, and 0.62 to 0.73, respectively.

## 4. Discussion

This meta-analysis found that the meta-analytic pooled sensitivity and DOR were the highest for NMLIT with APHE, followed by washout appearance and enhancement similar to pretreatment. All three of these LR-TR viable features showed equivalently high pooled specificity for diagnosing viable HCC. There was a tendency for the pooled sensitivities of these features to be higher on MRI than on CT, although the difference was not statistically significant. A significant threshold effect was noted for enhancement similar to pretreatment. Study heterogeneity was substantial except for the pooled specificity of washout appearance, and the heterogeneity was significantly affected by the type of reference standard, MRI contrast agent, and image analysis method. Given that LRT can be an effective alternative to surgical resection, especially for a subset of early-stage HCC [27], these results can provide valuable information for a wide range of clinicians involved in the management of HCC patients.

In accordance with previous studies assessing the performance of LR-TR [12,14,18,22], NMLIT with APHE was the most predictive of the viability of HCC among the three LR-TR viable features. In addition, NMLIT with APHE was the most frequently observed feature when evaluating treated observations (mean, 38.6%), indicating that the classification of treated observations as LR-TR viable was driven mainly by APHE. Therefore, the detection of NMLIT with APHE is of utmost importance during the treatment response evaluation of HCC following LRT. In the subgroup analysis results, APHE in MRI was more sensitive than and equivalently specific to CT in the prediction of the viability of HCC. This result is consistent with a previous study that reported HBA-enhanced MRI to be more sensitive than CT in evaluating tumor viability with the LR-TR algorithm [12]. The modality-based difference in the detection of viability is primarily due to beam-hardening artifacts and the parenchymal accumulation of iodized oil that occurs after conventional TACE, which is the most commonly used type of LRT [13,28,29]. These phenomena may mask APHE in viable tumors on CT but are not encountered when using MRI. Therefore, MRI is more useful than CT to detect APHE after TACE, and subtraction imaging can be especially helpful in HBA-enhanced MRI [14].

NMILT with washout appearance was also a useful imaging feature in predicting the viability of HCC, with moderate sensitivity and high specificity. Considering that some early-stage HCCs (17.3–31.6%) may not exhibit APHE [30], the washout appearance has incremental value in determining the viability of hypovascular HCC after LRT. However, there is room for further improvement of the sensitivity of the washout appearance. In HBA-enhanced MRI, the washout appearance can be determined only in the portal-venous phase according to the LI-RADS guidelines [7]. However, recent studies showed that the application of certain ancillary features, such as transitional or hepatobiliary phase hypointensity (i.e., extended washout [31]), significantly increased sensitivity without sacrificing specificity of the LR-TR algorithm [11,23]. Therefore, adopting these ancillary features may improve the moderate sensitivity of the washout appearance when using HBA-enhanced MRI to evaluate treatment response.

The pooled sensitivity of NMLIT with enhancement similar to pretreatment was unacceptably low (21% [95% CI, 6–53]) despite its high pooled specificity. The prevalence rate of this feature in treated observations was also reported to be low (mean, 18.2%) and variable (range, 0–60.2%). In addition, there was a considerable threshold effect (correlation coefficient, 0.64) that occurred when different thresholds or cutoff values were used to determine a positive test result [17]. Thus, there is some uncertainty about how to interpret the definition of NMLIT with enhancement similar to pretreatment presented in the LR-TR algorithm [7]. Another issue is how to evaluate this feature if the pre- and post-treatment imaging modalities are different (CT versus MRI). More specific definitions and clinical examples to illustrate the feature would be helpful for improving a low sensitivity and any interpretation discrepancies between image reviewers.

Meta-regression analyses revealed that the type of reference standard and MRI contrast agent were significant factors influencing study heterogeneity for NMLIT with APHE. Among the included studies, five used CCRS as a reference standard for viability, such as imaging follow-up, accumulation of ethiodized oil in the treated observation on post-TACE imaging, or concordance between contrast-enhanced ultrasound and MRI [11,13,21,24,26]. These results can lead to a lack of independence between diagnostic and reference standard tests, potentially overestimating diagnostic performance [32]. Indeed, studies using CCRS as a reference standard demonstrated significantly higher sensitivity (91% versus 63%) than those using only pathological reference standards. In addition, studies that used ECA showed a higher sensitivity and specificity of NMLIT with APHE than those using only HBA. These findings could be explained given the known challenges of HBA-enhanced MRI, including motion artifacts in the arterial phase and weaker APHE than ECA-enhanced MRI [33,34]. Therefore, the use of ECA rather than HBA-enhanced MRI as an imaging test after LRT may be more useful, but further studies are needed to validate this conjecture. Meanwhile, the image analysis method was significantly associated with the heterogeneity for the NMLIT with washout appearance. Considering that consensus-based decision making rarely reflects clinical practice in general [35], the method of image analysis should be considered when interpreting the results of individual study.

This meta-analysis has several limitations. Firstly, most of the studies were retrospective in design, and the number of studies reporting the performance of NMLIT with enhancement similar to pretreatment was small (*n* = 6). Secondly, because five studies used various types of LRT and did not separately report the performance of LR-TR viable features according to the LRT type, meta-analytic accuracy of each LRT type could not be evaluated; a meta-analysis using individual participant data is needed. Thirdly, substantial heterogeneity between studies limited the generation of robust meta-analytic estimates for diagnostic accuracy. To minimize this limitation, we investigated its sources and found that reference standards, MRI contrast agent, image analysis method, and threshold effect were related to study heterogeneity. Fourthly, 7 out of the 10 included studies were conducted in South Korea, an area where hepatitis B is endemic and LRT is widely used, thereby potentially limiting the generalizability of our results.

## 5. Conclusions

NMLIT with APHE provided the highest sensitivity and DOR for diagnosing viable HCC following LRT, while enhancement similar to pretreatment showed suboptimal performance. All these features showed equivalently high specificity for diagnosing the viability of HCC following LRT. Further refinement of the definition for enhancement similar to pretreatment may be necessary to improve the low sensitivity.

## Figures and Tables

**Table 1:** Characteristics of included articles.

Author (Publication Year)	Study Design	Number of Patients	Patient Age, Years *	Dominant Etiology of Liver Disease	No. of Treated Observations	Type of Locoregional Treatment	Imaging Modality	MRI Magnet	MRI Contrast Agent	Image Analysis	No. of Reviewers (Years of Experience)	Reference Standards for Viable HCC
Saleh TY (2019) [21]	Prospective	30	62.6 (49–72), mean (range)	Chronic viral hepatitis	41	TACE (100%)	MRI	3.0-T	ECA	NA	NA	CCRS ^†^
Kim SW (2020) [11]	Retrospective	183	59.9 ± 10.8	Hepatitis B	183	TACE (72.1%), RFA (23.0%), or DEB-TACE	MRI	1.5- or 3.0-T	HBA	Multiple reviewers with consensus	2 (5, 7 years)	Pathology or CCRS ^†^
Seo N (2020) [22]	Retrospective	114	54.0 ± 6.9	Hepatitis B	206	TACE (78.6%), RFA (16.5%), or DEB-TACE	CT (*n* = 113) or MRI (*n* = 53)	1.5- or 3.0-T	HBA or ECA	Multiple independent reviewers	2 (16, 17 years)	Pathology (explant)
Park S (2020) [23]	Retrospective	138	58 ± 9	Hepatitis B	138	TACE (66.7%), RFA or PEIT (13.0%)	CT (*n* = 138) and MRI (*n* = 138)	1.5- or 3.0-T	HBA	Multiple reviewers with consensus	2 (5, 7 years)	Pathology (explant or resection)
Bae JS (2021) [12]	Retrospective	165	62 ± 9	Hepatitis B	237	TACE (67.5%), RFA (22.0%), or PEIT (4.6%)	CT (*n* = 165) and MRI (*n* = 165)	1.5- or 3.0-T	HBA	Multiple independent reviewers	3 (7, 9, 14 years)	Pathology (explant)
Granata V (2021) [24]	Retrospective	64	74 (62–83), median (range)	Hepatitis B	136	RFA (72.1%) or MWA (27.9%)	MRI	1.5-T	ECA	Multiple reviewers with consensus	3 (NA)	CCRS ^†^
Huh J (2021) [13]	Retrospective	115	65.5 ± 10.4	Hepatitis B	151	TACE (100%)	CT	NA	NA	Multiple independent reviewers	2 (>7 years)	Pathology or CCRS ^†^
Mahmoud BE (2021) [26]	Retrospective	45	58.6 (45–74), mean (range)	NA	51	MWA (100%)	MRI	1.5-T	ECA	Multiple reviewers with consensus	3 (9, 11, 12 years)	CCRS ^†^
Yoon J (2021) [25]	Retrospective	27	55.9 ± 9.1	Hepatitis B	34	TARE (100%)	CT (*n* = 10) or MRI (*n* = 17)	3.0-T	HBA or ECA	Multiple reviewers with consensus	3 (2, 5, 9 years)	Pathology (explant or resection)
Youn SY (2021) [14]	Retrospective	90	57 (38–84), mean (range)	Hepatitis B	105	TACE (57.0%), RFA (23.8%), or DEB-TACE	MRI	1.5- or 3.0-T	HBA	Multiple reviewers with consensus	2 (6, 9 years)	Pathology (explant or resection)

* Unless otherwise stated, data are mean value, and data in parentheses are standard deviation. ^†^ Viable tumors are diagnosed by imaging follow-up, lipiodol accumulation in target observation following TACE, or concordance between contrast-enhanced ultrasound and MRI. MRI, magnetic resonance imaging; HCC, hepatocellular carcinoma; TACE, transarterial chemoembolization; ECA, extracellular contrast agent; NA, not available; CCRS, composite clinical reference standard; RFA, radiofrequency ablation; DEB, drug-eluting beads; HBA, hepatobiliary contrast agent; CT, computed tomography; PEIT, percutaneous ethanol injection therapy; MWA, microwave ablation.

**Table 2:** Meta-analytic summary estimates of imaging features for LR-TR viable category.

Imaging Feature	No. of Studies	No. of Observations		Summary Estimates				*p* for Publication Bias
			Sensitivity (95% CI)	*I^2^*	Specificity (95% CI)	*I^2^*	DOR (95% CI)	
Overall								
NMLIT with APHE	10	1153	81% (63–92)	93%	95% (88–98)	92%	81 (25–261)	0.15
NMLIT with washout appearance	8	1068	55% (34–75)	92%	96% (94–98)	32%	32 (13–82)	0.54
NMLIT with enhancement similar to pretreatment	6	709	21% (6–53)	96%	98% (92–100)	79%	14 (5–39)	0.44
MRI								
NMLIT with APHE	8	968	87% (67–96)	96%	93% (84–97)	93%	97 (23–408)	0.16
NMLIT with washout appearance	6	883	66% (41–84)	91%	95% (93–97)	25%	39 (12–123)	0.51
NMLIT with enhancement similar to pretreatment	4	485	32% (16–54)	92%	96% (89–99)	59%	12 (5–28)	0.49
CT								
NMLIT with APHE	4	729	50% (36–64)	91%	95% (89–98)	80%	19 (11–35)	0.93
NMLIT with washout appearance	4	729	35% (17–59)	96%	97% (93–99)	54%	19 (9–41)	0.29
NMLIT with enhancement similar to pretreatment	4	689	15% (1–68)	97%	99% (87–100)	85%	12 (3–41)	0.98

LR-TR, Liver Imaging Reporting and Data System treatment response; CI, confidence interval; DOR, diagnostic odds ratio; NMLIT, nodular, mass-like, or irregular thick tissue in or along the treated lesion; APHE, arterial phase hyperenhancement; MRI, magnetic resonance imaging; CT, computed tomography.

**Table 3:** Meta-regression analysis of accuracy for imaging features of LR-TR viable category.

Imaging Feature	Covariates	Sensitivity (95% CI)	Specificity (95% CI)	*p*
NMLIT with APHE	Reference standard for viable HCC			0.02
	Pathology only	63% (43, 84)	94% (87, 100)	
	CCRS only or both	91% (82, 100)	95% (90, 100)	
	MRI contrast agent			0.03
	Hepatobiliary agent	73% (50, 97)	89% (81, 97)	
	Extracellular agent or both	90% (78, 100)	97% (95, 100)	
	Type of LRT			0.72
	Transcatheter therapy (>70%) *	85% (68, 100)	93% (85, 100)	
	Others ^†^	77% (54, 99)	96% (92, 100)	
	Image analysis			0.23
	Multiple independent reviewers	62% (38, 85)	96% (91, 100)	
	Multiple reviewers with consensus	85% (73, 98)	94% (86, 100)	
	Percentage of viable HCC			0.23
	≥50%	67% (39, 94)	94% (85, 100)	
	<50%	88% (76, 100)	95% (90, 100)	
NMLIT with washout appearance	Reference standard for viable HCC			0.71
	Pathology only	49% (23, 76)	96% (93, 99)	
	CCRS only or both	66% (32, 99)	96% (94, 99)	
	MRI contrast agent			0.59
	Hepatobiliary agent	57% (33, 81)	95% (93, 98)	
	Extracellular agent or both	68% (39, 97)	97% (94, 100)	
	Type of LRT			0.23
	Transcatheter therapy (>70%) *	41% (15, 67)	96% (93, 98)	
	Others ^†^	68% (43, 92)	97% (95, 99)	
	Image analysis			0.03
	Multiple independent reviewers	34% (16, 53)	97% (95, 99)	
	Multiple reviewers with consensus	73% (56, 91)	95% (92, 98)	
	Percentage of viable HCC			0.75
	≥50%	52% (21, 82)	95% (91, 99)	
	<50%	59% (28, 90)	97% (95, 98)	
NMLIT with enhancement similar to pretreatment	MRI contrast agent			0.20
	Hepatobiliary agent	42% (19, 65)	95% (90, 100)	
	Extracellular agent or both	24% (1, 47)	100 (100, 100)	
	Type of LRT			0.08
	Transcatheter therapy (>70%) *	9% (0, 24)	100% (100, 100)	
	Others ^†^	42% (4, 79)	95% (89, 100)	
	Image analysis			0.24
	Multiple independent reviewers	13% (0, 28)	98% (96, 100)	
	Multiple reviewers with consensus	52% (8, 95)	94% (86, 100)	
	Percentage of viable HCC			0.20
	≥50%	37% (8, 67)	97% (93, 100)	
	<50%	6% (0, 18)	98% (96, 100)	

* Studies in which transarterial chemo- or radioembolization was performed in more than 70% of observations. ^†^ Studies in which transcatheter therapy was performed in less than 70% of observations or only ablation therapy was performed. LR-TR, Liver Imaging Reporting and Data System treatment response; CI, confidence interval; NMLIT, nodular, mass-like, or irregular thick tissue in or along the treated lesion; APHE, arterial phase hyperenhancement; HCC, hepatocellular carcinoma; CCRS, composite clinical reference standard; MRI, magnetic resonance imaging; LRT, locoregional treatment.

## Data Availability

All data accessed and analyzed in this study are available in the article and its Supplementary Materials.

## Supplementary Materials

The following are available online at https://www.mdpi.com/article/10.3390/cancers13174432/s1, Table S1: Search queries, Table S2: Numbers of true positives, false positives, false negatives, and true negatives of each imaging feature of LR-TR viable category for diagnosing viable HCC, Figure S1: results of quality assessments of the articles according to QUADAS-2 criteria, Figure S2: hierarchical summary receiver operating characteristic curves for the accuracy of arterial phase hyperenhancement (a), washout appearance (b), or enhancement similar to pretreatment (c), Figure S3: Deeks’ funnel plot to evaluate publication bias regarding arterial phase hyperenhancement (a), washout appearance (b), or enhancement similar to pretreatment (c).
