# Supplementary material for: Per-Feature Accuracy of Liver Imaging Reporting and Data System Locoregional Treatment Response Algorithm: A Systematic Review and Meta-Analysis
Source: Cancers (Basel). 2021 Sep 2;13(17):4432. doi: 10.3390/cancers13174432 (PMC8430492; doi:10.3390/cancers13174432)
Supplement: Supplementary file 1 [file cancers-13-04432-s001.zip › cancers-1332751-supplementary.pdf]

cancers

Supplementary Materials: Per-Feature Accuracy of Liver Imaging Reporting and Data System Locoregional Treatment Response Algorithm: A Systematic Review and Meta-Analysis

Yeon Jong Huh, Dong Hwan Kim, Bohyun Kim, Joon-Il Choi and Sung Eun Rha

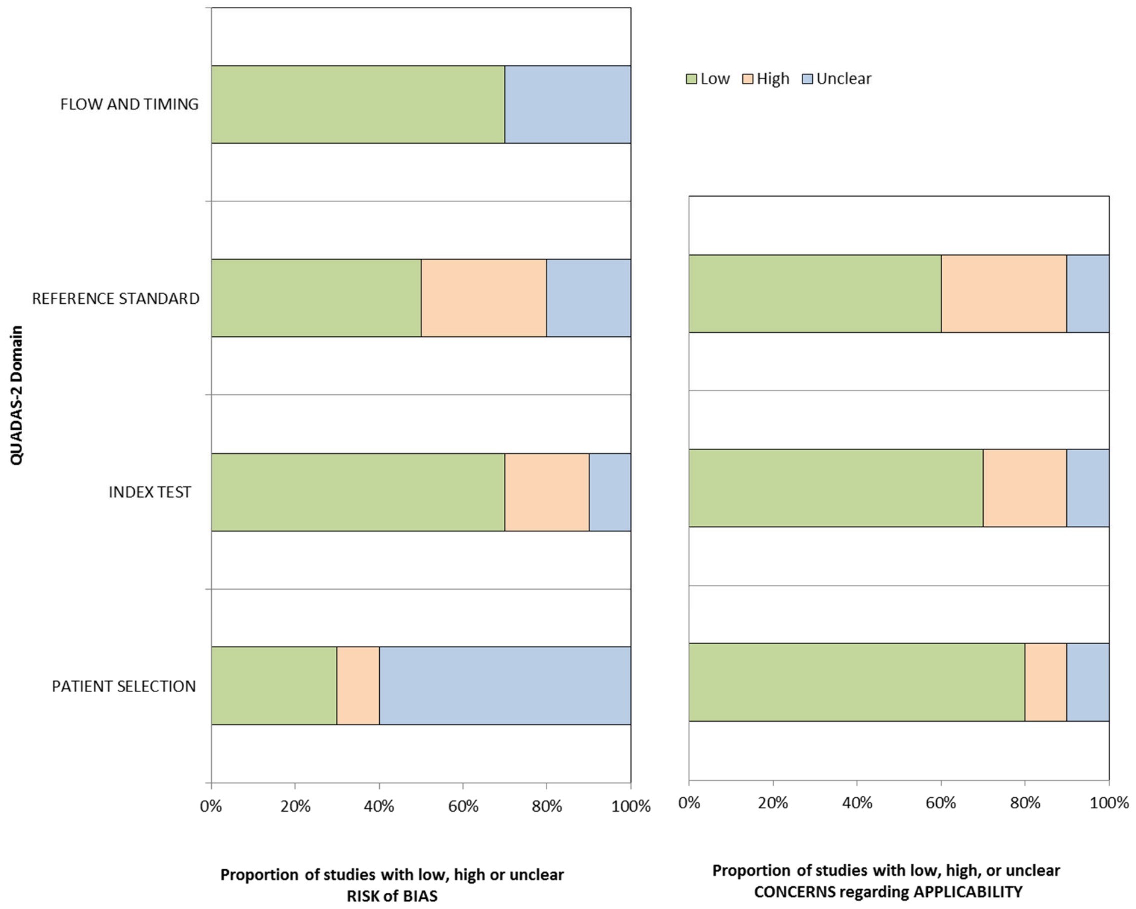

Figure S1. Results of quality assessments of the articles according to QUADAS-2 criteria. The methodological quality distribution of the articles is presented as the proportions of articles (0–100%) with a low (i.e., high quality), high, and unclear risk of bias and the proportions of articles with low (i.e., high quality), high, or unclear concerns regarding applicability for each domain.

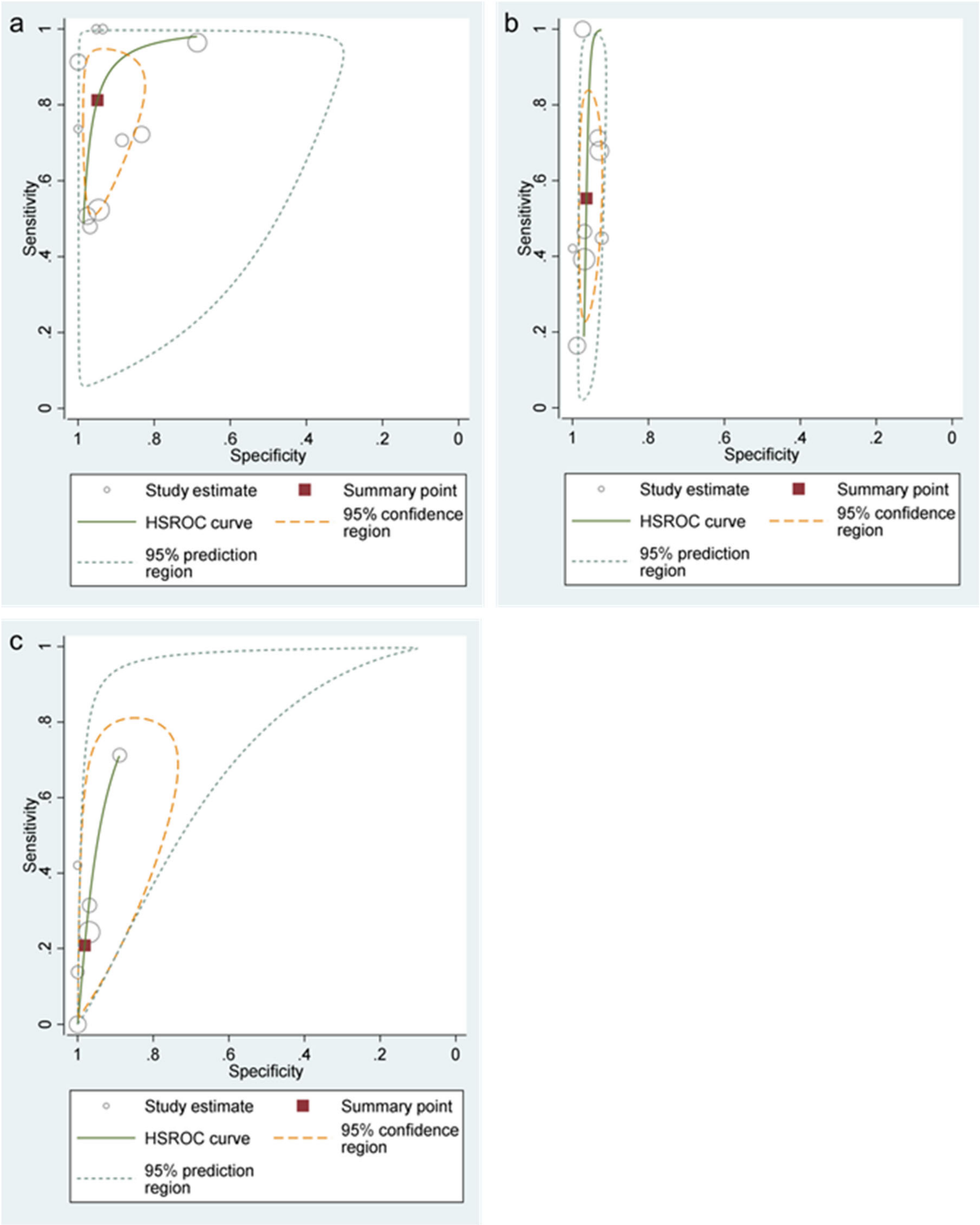

Figure S2. Hierarchical summary receiver operating characteristic curves for the accuracy of arterial phase hyperenhancement (**a**), washout appearance (**b**), or enhancement similar to pretreatment (**c**).

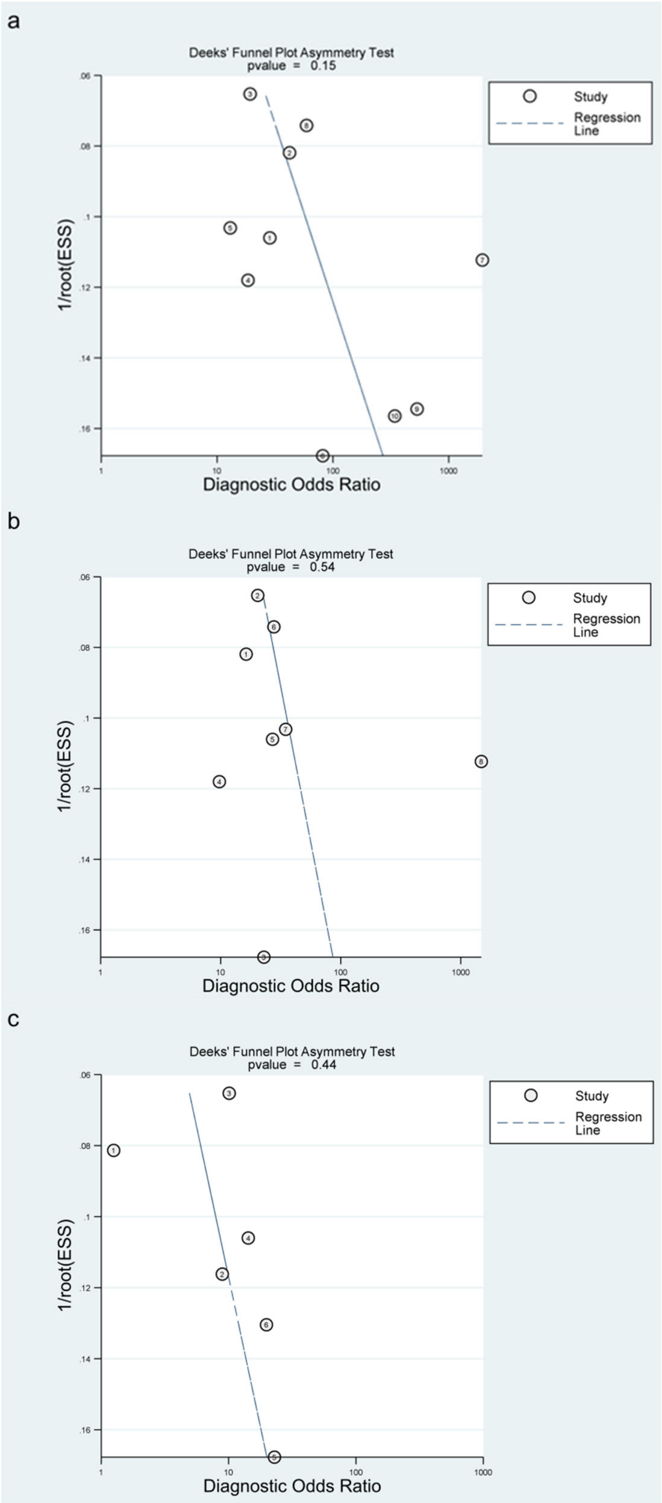

Figure S3. Deeks' funnel plot to evaluate publication bias regarding arterial phase hyperenhancement (**a**), washout appearance (**b**), or enhancement similar to pretreatment (**c**).

Table S1. Search queries.

No.	Search Queries for MEDLINE
#1	("Liver Neoplasms"[Mesh] OR "Liver"[Mesh]) AND "Radiology Information Systems"[mesh]
#2	"LI-RADS"[TW] OR "LR-TR"[TW] OR "liver imaging reporting"[TW] OR "LI-RADS Treatment Response"[TW]
#3	#1 OR #2
#4	Magnetic Resonanc*[TW] OR MRI[TW] OR MR[TW] OR CT[TW] OR Computed Tomography[TW]
#5	#3 AND #4
#6	#5 AND English[Lang] AND ("2017/01/01"[PDAT]: "3000/12/31"[PDAT])
No.	Search queries for EMBASE
#1	'liver imaging reporting and data system'/exp
#2	('LI-RADS' OR 'LR-TR' OR 'liver imaging reporting' OR 'LI-RADS Treatment Response'):ab,ti,kw
#3	#1 OR #2
#4	('Magnetic Resonanc*' OR MRI OR MR OR CT OR 'Computed Tomography'):ab,ti,kw
#5	#3 AND #4
#6	#5 AND [english]/lim AND [2017-2021]/py

Table S2. Numbers of true positives, false positives, false negatives, and true negatives of each imaging feature of LR-TR viable category for diagnosing viable HCC.

Imaging Feature	Author (Publication Year)	Total Number of Observations	Number of Observations			
			TP	FP	FN	TN
NMLIT with APHE	Saleh TY (2019)	40	19	1	0	20
	Kim SW (2020)	183	81	31	3	68
	Seo N (2020)	84	41	3	17	23
	Park S (2020)	138	78	5	30	25
	Bae JS (2021)	237	56	7	51	123
	Granata V (2021)	136	21	0	2	113
	Huh J (2021)	151	34	2	33	82
	Mahmoud BE (2021)	45	14	2	0	29
	Yoon J (2021)	34	14	0	5	15
	Youn SY (2021)	105	35	1	38	31
NMLIT with washout appearance	Kim SW (2020)	183	57	7	27	92
	Seo N (2020)	84	26	2	32	24
	Park S (2020)	138	77	2	31	28
	Bae JS (2021)	237	42	4	65	126
	Granata V (2021)	136	23	3	0	110
	Huh J (2021)	151	11	1	56	83
	Yoon J (2021)	34	8	0	11	15
	Youn SY (2021)	105	34	1	39	31
NMLIT with enhancement similar to pretreatment	Seo N (2020)	84	8	0	50	26
	Park S (2020)	98	57	2	23	16
	Bae JS (2021)	237	26	4	81	126
	Huh J (2021)	151	0	0	67	84
	Yoon J (2021)	34	8	0	11	15
	Youn SY (2021)	105	23	1	50	31

LR-TR, Liver Imaging Reporting and Data System treatment response; HCC, hepatocellular carcinoma; NMLIT, nodular, mass-like, or irregular thick tissue in or along the treated lesion; APHE, arterial phase hyperenhancement; TP, true positive; FP, false positive; FN, false negative; TN, true negative.
